# Prophylactic melatonin significantly reduces Alzheimer’s neuropathology and associated cognitive deficits independent of antioxidant pathways in AβPP^swe^/PS1 mice

**DOI:** 10.1186/s13024-015-0027-6

**Published:** 2015-07-11

**Authors:** G. O’Neal-Moffitt, V. Delic, P. C. Bradshaw, J. Olcese

**Affiliations:** Department of Biomedical Sciences, College of Medicine, Florida State University Program in Neuroscience, 1115 West Call Street, Tallahassee, FL 32306 USA; Department of Cell Biology, Microbiology, and Molecular Biology, University of South Florida, 4202 East Fowler Avenue, ISA2015, Tampa, FL 33620 USA

**Keywords:** Melatonin, Alzheimer’s, Melatonin receptors, Cognition, Mice, Cytochrome c oxidase

## Abstract

**Background:**

Alzheimer’s disease (AD) underlies dementia for millions of people worldwide, and its occurrence is set to double in the next 20 years. Currently, approved drugs for treating AD only marginally ameliorate cognitive deficits, and provide limited symptomatic relief, while newer substances under therapeutic development are potentially years away from benefiting patients. Melatonin (MEL) for insomnia has been proven safe with >15 years of over-the-counter access in the US. MEL exerts multiple complementary mechanisms of action against AD in animal models; thus it may be an excellent disease-modifying therapeutic. While presumed to provide neuroprotection via activation of known G-protein-coupled melatonin receptors (MTNRs), some data indicate MEL acts intracellularly to protect mitochondria and neurons by scavenging reactive oxygen species and reducing free radical formation. We examined whether genetic deletion of MTNRs abolishes MEL’s neuroprotective actions in the AβPP^swe^/PSEN1dE9 mouse model of AD (2xAD). Beginning at 4 months of age, both AD and control mice either with or without both MTNRs were administered either MEL or vehicle in drinking water for 12 months.

**Results:**

Behavioral and cognitive assessments of 15-month-old AD mice revealed receptor-dependent effects of MEL on spatial learning and memory (Barnes maze, Morris Water Maze), but receptor-independent neuroprotective actions of MEL on non-spatial cognitive performance (Novel Object Recognition Test). Similarly, amyloid plaque loads in hippocampus and frontal cortex, as well as plasma Aβ_1–42_ levels, were significantly reduced by MEL in a receptor-independent manner, in contrast to MEL’s efficacy in reducing cortical antioxidant gene expression (Catalase, SOD1, Glutathione Peroxidase-1, Nrf2) only when receptors were present. Increased cytochrome c oxidase activity was seen in 16mo AD mice as compared to non-AD control mice. This increase was completely prevented by MEL treatment of 2xAD/MTNR+ mice, but only partially prevented in 2xAD/MTNR- mice, consistent with mixed receptor-dependent and independent effects of MEL on this measure of mitochondrial function.

**Conclusions:**

These findings demonstrate that prophylactic MEL significantly reduces AD neuropathology and associated cognitive deficits in a manner that is independent of antioxidant pathways. Future identification of direct molecular targets for MEL action in the brain should open new vistas for development of better AD therapeutics.

## Background

Current therapeutics and drugs for improving memory loss in Alzheimer’s disease (AD) patients only marginally ameliorate cognitive deficits, and provide patients with only restricted symptomatic relief [1–3]. Many compounds under therapeutic development are years away from benefiting AD patients. Thus, there is a critical need for rapid development of safe, effective therapeutics against AD. Melatonin analogs may provide such a therapeutic.

Melatonin (MEL) is known to modulate many physiological functions [4–6]. With advancing age and certain age-related diseases, the endogenous secretion of MEL drops markedly, and MEL supplementation can ameliorate sleep disorders in the aged [7]. Declines in blood and CSF MEL levels in Alzheimer disease patients have been reported to parallel the progression of neuropathology [8, 9]. Although MEL has been given routinely to AD patients to suppress sundowning, few publications have investigated the cognitive effects of MEL administration on AD patients. MEL stabilized cognitive function in AD patients over a 2–3 year period [10] and improved cognitive performance in mild cognitive impairment (MCI) individuals [11]. A 3-year course of MEL treatment to one of a pair of monozygotic AD twins resulted in milder cognitive impairment for the treated twin [12]. Despite these epidemiologic and anecdotal reports, all of which involved low MEL doses (≤9 mg/day), no controlled clinical studies of MEL effects on cognition in AD patients have been published. MEL is amphiphilic, thus it is able to penetrate all cellular compartments and freely enter the brain, especially from the CSF [13, 14]. The pharmacokinetics and oral bioavailability of exogenously-administered MEL and analogs have been well-established in preclinical and clinical studies [15].

Considerable *in vitro* evidence supports the premise that MEL exerts an anti-amyloid-β (Aβ) aggregation effect [16–18]. MEL has been shown to protect against Aβ-induced neurotoxicity *in vitro* and *in vivo* [17–22]. We and others have also demonstrated significantly reduced amyloid plaque burden in AD mice treated for several months [23, 24]. Interestingly, the neuroprotection afforded by MEL in AD mice appears to be age-dependent [25] in as much as treating mice from 4 to 8 months was not found to be significantly beneficial, whereas MEL from 8 to 12 months of age (or from 4 to 12 months of age) significantly preserved cognition while reducing amyloid plaque load in these animals. In another study [26], a very low dose of MEL (0.08 mg/day) was administered to aged Tg2576 (APP^swe^) mice beginning at 14–18 months of age. Neither soluble Aβ levels nor Aβ deposition was affected in cortex, leading the authors to conclude that MEL is unlikely to be a treatment for already established AD. However, the low dose of MEL utilized in their study, the very late onset of treatment, the lack of cognitive evaluation, and non-assessment of any other key markers, are clearly in contrast to our previous studies and the present study.

MEL has often been reported to have anti-inflammatory (and occasionally pro-inflammatory) properties in many species, including humans [27–29]. It is noteworthy that MEL administration lessens Aβ-induced pro-inflammatory cytokine levels in rat and mouse brains [23, 30]. Indeed, MEL may represent a new class of anti-inflammatory agent [31], with accumulating evidence for a significant role in reducing neuroinflammation via diverse mechanisms (Hardeland et. 2015 ibid). Melatonin offers neuroprotection at the level of mitochondrial function [32, 33]. Consistent with this idea, our published work points to reduced oxidative stress in a mouse model of AD (AβPP^swe^/PSEN1dE9) after administration of MEL for ≥ 1 month [23, 33] and a MEL-mediated decrease in COX activity in the striatum of our double AD mice. Finally, evidence demonstrates that MEL can decrease tau hyperphosphorylation in cell cultures [34]. Another mechanism through which MEL may protect against cognitive impairment is through stabilization and enhancement of dendritic structure. Prior studies have shown that MEL is capable of preventing loss of dendritic length and number for pre-frontal cortical neurons of rats subjected to global ischemia [35–37]. MEL has also been reported to promote dendritogenesis in the hippocampus [38]. Thus, MEL appears to exert multiple complementary mechanisms of action in the brain and hence may be an excellent therapeutic against AD.

Despite these consistent and significant actions of MEL on the cognition and pathology of the AD mouse brain, the mechanisms of MEL action remain unclear. In two recent reports [39, 40] the cognitive function of transgenic AD mice was assessed after treatment with the specific, nonselective MTNR ligand, Ramelteon® (Takeda Pharmaceuticals), for up to 6 months. Ramelteon® is a commercially available, clinically tested (for insomnia), highly specific agonist at both MTNRs, having no direct intracellular activity [41]. Intriguingly, and despite evidence for reduced hippocampal protein oxidation [40], Ramelteon® in these two studies was ineffective in lowering amyloid plaque load or preserving cognitive functions. The most parsimonious interpretation of these findings is that MEL acts via MTNR-independent mechanisms to cognitively protect the amyloid-afflicted brain.

In an effort to provide clarity on this matter, we have generated transgenic AβPP^swe^/ PSEN1dE9 (2xAD) mice that lack both known MTNRs (MTNR-) in order to determine whether MEL has neuroprotective capabilities that are independent of these receptors. Cognitive performance, Aβ hippocampal plaque load, blood Aβ_1–40_ and Aβ_1–42_ levels, and key markers of brain oxidative stress were assessed in 2xAD mice with or without MTNRs that received long-term MEL. Compared to NonAD mice, the 2xAD animals developed significant cognitive deficits that were lessened by MEL. In some cognitive domains this neuroprotection was seen even in the absence of MTNRs. Similarly, amyloid plaque load in the hippocampus and frontal cortex as well as circulating levels of Aβ_1–42_ were significantly lowered by MEL in an MTNR-independent manner. These results strongly suggest that MEL provides neuroprotective effects in the AD brain in a manner that is to some degree independent of the two known membrane receptors.

## Results

### Behavioral testing

#### Sensorimotor and locomotor behavior

**Fig. 1:**
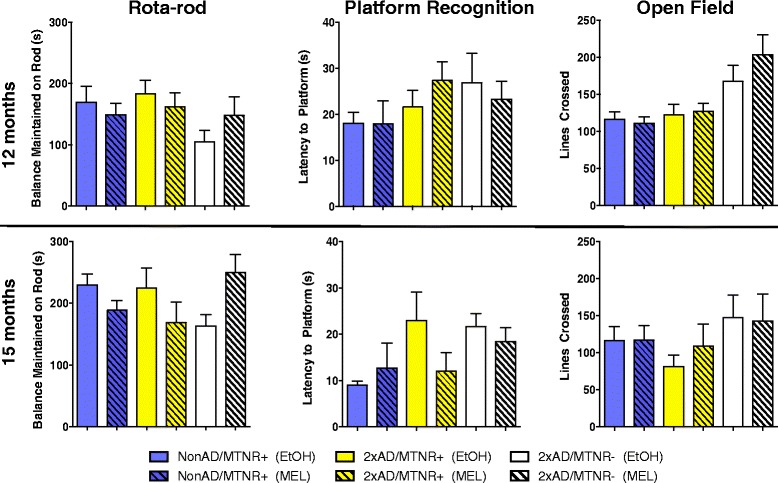
Behavioral assessment of sensorimotor function and activity in 12- and 15-month-old mice treated either with melatonin (MEL) or vehicle (EtOH). The Rota-rod revealed no significant difference at any age in the length of time balance was maintained on a rotating horizontal rod that accelerated from 4 to 40 rpm. A test of visual and motor skills in a water maze Platform Recognition test revealed no significant differences. Animals in both groups were able to see, swim to, and climb onto the platform. Locomotor activity was assessed with an Open Field test. No significant differences were found in control (NonAD) or 2xAD mice either with melatonin receptors (MTNR+) or without melatonin receptors (MTNR-). All tests were evaluated with 2-way ANOVA; 12-month *n* = 11–14; 15-month *n* = 4–7; error bars represent SEM; *p* > 0.05

No evidence of sensorimotor deficits was seen on the Platform Recognition or Rota-rod tests at any of the ages tested (Fig. 1). There were no statistical differences between groups, trials or the interaction in the Platform Recognition test and no significant differences (*p* > 0.05) on Rota-rod performance between the two genotypes at any age. It is worth noting that these mice clearly had no visual deficits, despite their C3H background, most likely because they were out crossed for multiple generations to the C57BL/6 line.

Assessment of locomotor activity was performed using the Open Field test. No statistically significant differences in the number of lines crossed per trial were seen at 12-months of age, even though 2xAD mice on the MTNR- background crossed more lines than mice with MTNRs. By 15-months of age these differences are largely attenuated as was recently described for NonAD/MTNR- mice [42].

##### Anxiety behavior

**Fig. 2:**
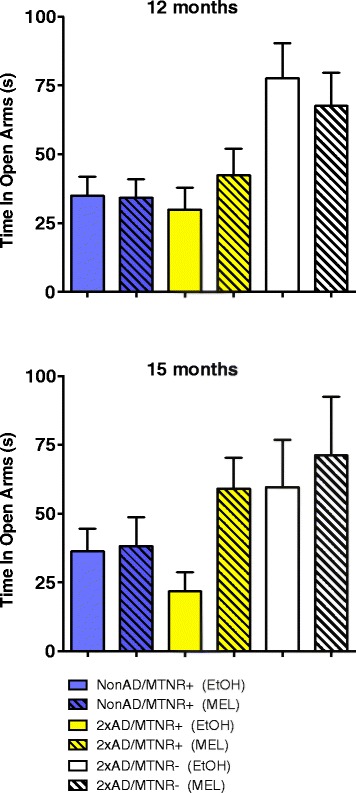
Determination of anxiety levels by time in spent in the open arms of the Elevated Plus Maze. No significant differences were seen in either the 12-month-old or in the 15-month-old NonAD control or 2xAD mice, irrespective of the whether they possessed melatonin receptors (MTNR+) or lacked them (MTNR-). Evaluated with 2-way ANOVA; 12-month *n* = 10-13; 15-month *n* = 4–7; error bars represent SEM; *p* > 0.05

Determination of anxiety/emotionality was performed using the Elevated Plus Maze, which incorporates both open and closed arms, the mice generally preferring the closed arms. Thus, the greater the time spent in the open arms is considered a measure of decreased anxiety. As seen in Fig. 2, the NonAD and 2xAD mice at 12 and at 15 months of age spent similar amounts of time in the open arms, despite a clear trend to less anxiety (more time spent in the open arms) in the 2xAD/MTNR- mice at 12 months of age. Additionally, at 12 months of age, but even more apparent at 15 months, MEL tended to increase the time spent in the open arms, i.e. to reduce anxiety. However, this effect was seen only in MTNR+ mice. There were no significant group differences in the ratio of open arm entries to closed arm entries.

### Cognitive performance

#### Non-spatial cognitive: novel object recognition test

**Fig. 3:**
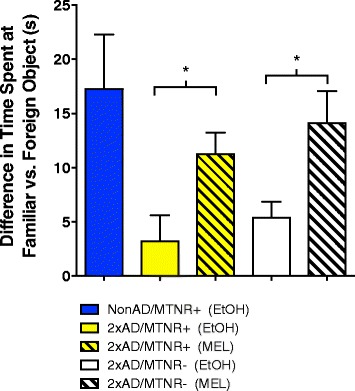
Measurement of non-spatial memory in 15-month-old mice with the novel object recognition test. This test evaluates the difference in time spent investigating a familiar versus foreign object. The vehicle-treated 2xAD mice (clear yellow and white bars) showed significantly poorer performance when compared to the NonAD/MTNR+ control mice with or without MEL. This was alleviated in the 2xAD mice that had received melatonin (MEL; striped bars), irrespective of the presence (MTNR+) or absence (MTNR-) of melatonin receptors. Evaluated with 2-way ANOVA; *n* = 4–7; error bars represent SEM; *p* < 0.05

The novel object recognition test (NORT) is a facile behavioral assay based on the spontaneous preference of rodents to interact more with an unfamiliar object than with a familiar one. As such there is little to no stress on the animal and it does not require spatial orientation. It has been used to assess deficits in learning and memory in various mouse models of AD [43, 44]. We tested the effects of MEL on performance in the NORT in 15 month-old AD mice with or without their MTNRs. As shown in Fig. 3, both groups of vehicle-treated 2×AD mice performed poorly, i.e. showing almost equal times exploring the novel objects and the familiar (only small differences between the two). In contrast, the 2×AD mice that were treated with MEL showed significantly improved performance levels that were comparable to the NonAD control level. These MEL effects were independent of the presence of the MTNRs. No effects of MEL were seen in NonAD mice.

#### Spatial cognitive: barnes maze and the morris water maze

**Fig. 4:**
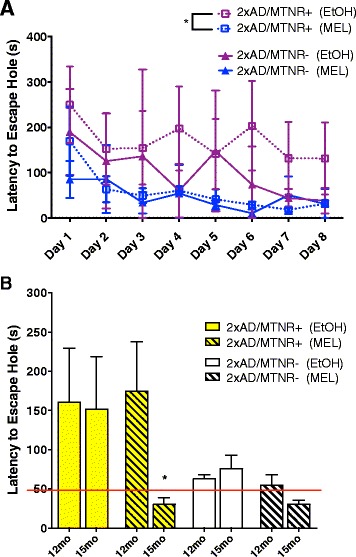
Spatial learning and memory in AD mice as determined with the Barnes Maze. **a** Latency to find the escape hole decreased over 8 days of testing in 15-month old 2xAD/MTNR+ mice that received MEL, but not in MEL-treated 2xAD/MTNR- mice. **b** Comparison of the average latency to escape on initial testing (days 1–4) for 12-month-old 2xAD mice as compared to the same mice at 15 months of age during final testing (days 5–8). Only the 2xAD/MTNR+ that received MEL exhibited significantly improved performance. Evaluated with 2-way RM ANOVA; *n* = 4–7; error bars represent SEM; *p* < 0.05

Mice were tested for cognitive performance with two standard behavioral tests for spatial/reference learning, the Barnes Maze (circular platform test) and the Morris Water Maze (the probe test). At 15 months of age the 2xAD/MTNR+ mice treated with MEL showed learning in the Barnes Maze by day 8 as compared to day 1 (Fig. 4a) in contrast to EtOH-treated controls. However, 2xAD/MTNR- mice were quicker in the Barnes maze in general, most likely due to their increased activity and generally improved cognition as reported previously. MEL treatment seemed to slightly potentiate this effect even in absence of the receptors.

Comparisons of the final latencies (days 5–8 of testing) to find the escape hole in the same mice at 12-month of age versus their recall at 15-months of age (Fig. 4b) revealed that vehicle-treated mice 2×AD were not able to recall and improve their performance after three months. In contrast, the MEL-treated 15-month-old 2×AD mice made significant improvements over their performance at 12-months, consistent with enhanced learning capabilities. The superior performance in the 2×AD/MTNR- mice was likely due to the consequences of genetic deletion of the MTNRs, which gives even NonAD/MTNR- mice a cognitive advantage over MTNR+ mice as we recently reported [42]. In both 2xAD groups MEL treatment enabled superior performance at 15 months when compared to 12 months, although in the 2xAD/MTNR- group there was only marginal further improvement, presumably due to a “floor effect”.

**Fig. 5:**
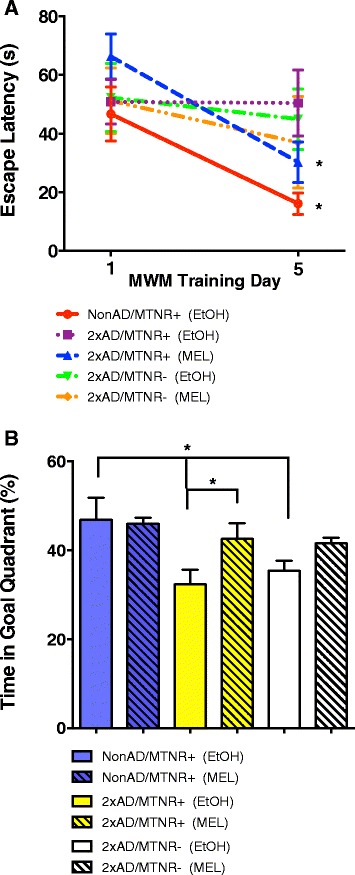
Assessment of spatial learning and memory in 15-month-old mice using the Morris Water Maze. **a** Averaged escape latency in the Morris Water maze, training day 1 versus training day 5, with four successive trials per day. NonAD/MTNR+ vehicle treated mice (red line) showed a significant reduction in their escape latency to the submerged platform by day 5. MEL-treated 2xAD/MTNR+ mice (blue line) also had significantly improved performance while none of the other Alzheimer mice showed any significant learning. **b** Probe test follow-up after Morris Water maze training as determined by percentage of time spent in the goal quadrant. Retention of the memory of the goal quadrant was significantly reduced in the 2xAD mice as compared to the NonAD control mice, irrespective of the presence or absence of melatonin receptors (clear yellow and white bars). Treatment with MEL significantly improved memory in the 2xAD/MTNR+ (yellow, striped bar), but not in the 2xAD/MTNR- mice. Evaluated with 2-way RM ANOVA; *n* = 4–7; error bars represent SEM; *p* < 0.05

The Morris Water Maze (Fig. 5a) provides for another assessment of spatial learning (shorter escape latencies with training) as well as testing for working memory (the probe test). In the case of escape latencies, clear improvements were seen in the NonAD/MTNR+ mice when comparing the first and last training days (P < 0.05). Neither of the vehicle-treated 2xAD groups showed any spatial learning. However, the 2xAD/MTNR+ mice that received MEL had similar rates of learning to the NonAD/MTNR+ control mice. Similar trends were seen with the probe test, where the 2xAD mice spent the least amount of time in the goal quadrant (Fig. 5b) while the NonAD/MTNR+ controls spent the most time. 2xAD/MTNR+ mice that received MEL had performance levels comparable to the NonAD/MTNR+ controls.

### Survival curve

**Fig. 6:**
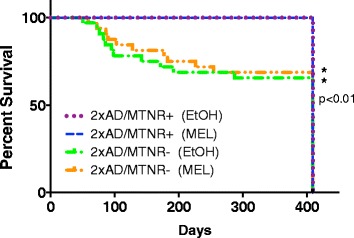
A Kaplan-Meier survival curve for the 2xAD mice over the course of the study. The mice with melatonin receptors (MTNR+) suffered no losses (*n* = 35), while the mice lacking melatonin receptors (MTNR-) had increasing mortality beginning at 70 days (from *n* = 62 only 41 survived). MEL or vehicle treatment had no effect on survival

In the early months of this study we noticed a significant degree of mortality in the 2xAD/MTNR- mice of our colony. A Kaplan-Meier survival plot (Fig. 6) revealed that within the first 3–9 months of age, nearly one-third of these animals were found dead in their cages, while no animals of the 2xAD/MTNR+ groups died. The mortality of the 2xAD/MTNR- mice was marginally, but not significantly, affected by MEL treatment. For this reason numerous cohorts of 2xAD/MTNR- mice had to be combined later in our study in order to attain sufficient animal numbers for behavioral and neuropathological studies.

### Immunohistochemistry

**Fig. 7:**
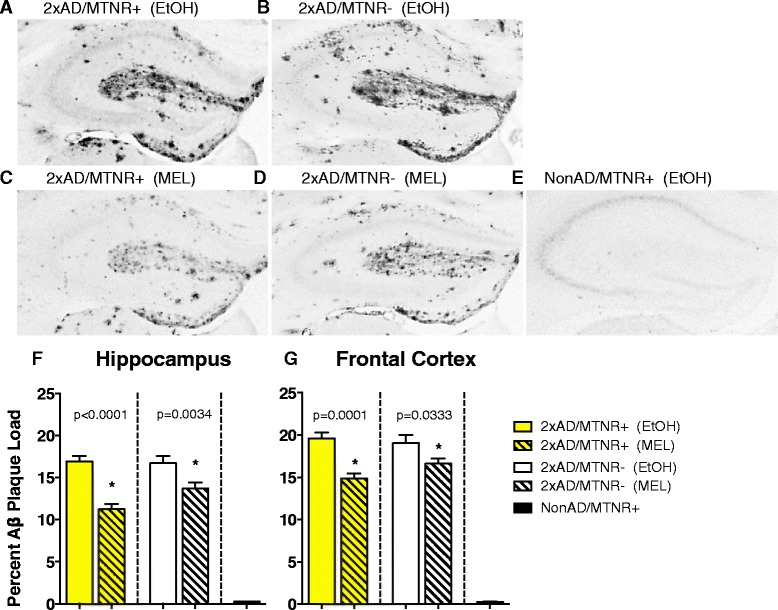
Immunohistochemical detection of amyloid plaques in the hippocampus of 15-month-old 2xAD mice treated with either MEL (**c**, **d**) or vehicle (**a**, **b**). NonAD mice did not develop plaques (E). Quantitative analysis of plaque load with ImageJ revealed significant MEL effects in both MTNR+ (*p* < 0.0001) and MTNR- (*p* = 0.0034) 2xAD hippocampi (**f**). Similar MEL effects were also seen in the frontal cortex (**g**) in both the 2xAD/MTNR+ mice (*p* = 0.0001) and in the 2xAD/MTNR- mice (*p* = 0.0333). Evaluated with 2-way ANOVA; *n* = 3–4; error bars represent SEM

Long-term MEL treatment induced clear reductions in amyloid beta deposition in 15-month-old 2xAD mice, as indexed by Aβ burden, in both the hippocampus (Fig. 7a-d) and the frontal cortex (Fig. 7g). NonAD brains presented with essentially no plaques (Fig. 7e). Quantification of Aβ plaque area (Fig. 7f,g) revealed substantial reductions of 33.5 % and 18 % in the brains of both 2×AD/MTNR+ and 2xAD/MTNR- mice, respectively, that received MEL treatment.

### ELISA for plasma Aβ peptides

**Fig. 8:**
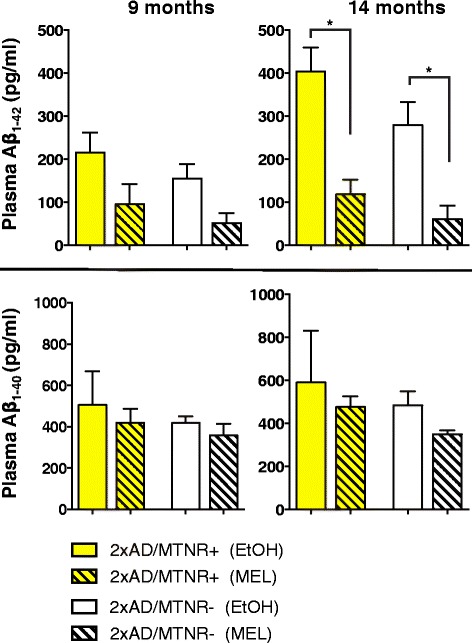
ELISA measurement of plasma Aβ_1–40_ and Aβ_1–42_ levels in 9-month-old and 14-month-old 2xAD mice. In all cases, MEL tended to lower amyloid peptide levels. A significant effect of MEL was seen at 14 months for Aβ_1–42_ levels in both MTNR+ mice (*p* = 0.0043) and in MTNR- mice (*p* = 0.011). Evaluated with 2-way ANOVA; *n* = 3–5; error bars represent SEM

Plasma levels of the amyloid peptides Aβ_1–40_ and Aβ_1–42_ were determined in cohorts of 2xAD mice at 9 months and 14 months of age (Fig. 8). While there were no significant differences in peptide levels between 2xAD animals possessing or lacking MTNRs, there were clear trends for reduced Aβ levels in the MEL-treated groups. In particular, MEL led to a significantly lowered serum Aβ_1–42_ level at 14 months of age, irrespective of the presence or absence of MTNRs.

### Cytochrome C oxidase activity

#### Melatonin receptors slightly delay the aging-induced loss of cytochrome c oxidase activity

**Fig. 9:**
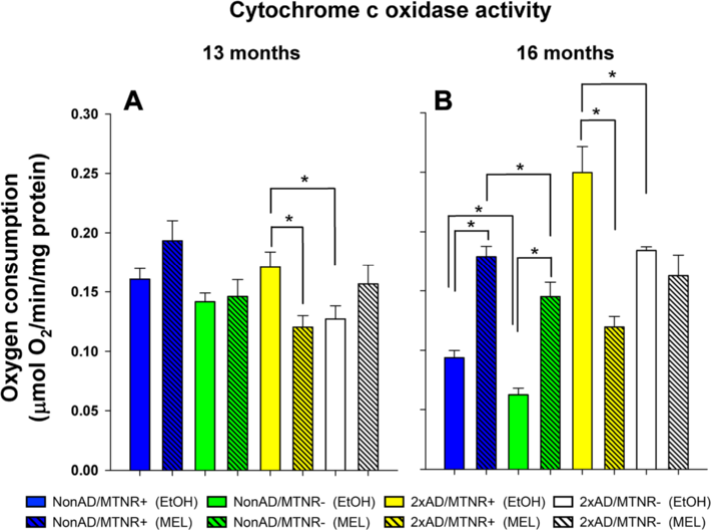
Complex IV activity in striatal extracts from 13- and 16-month mice. There was a decline in complex IV activity in NonAD mice and an increase in complex IV activity in AD mice from 13 to 16 months irrespective of MEL receptor status. **a** At 13 months 2xAD/MTNR+ mice had higher complex IV activity than 2xAD/MTNR- mice (*p* = 0.03). MEL treatment of the 2xAD/MTNR+ mice decreased complex IV activity (*p = 0.03). **b** At 16 months NonAD/MTNR+ mice had higher complex IV activity than NonAD/MTNR- mice (*p = 0.005) and 2xAD/MTNR+ mice had higher complex IV activity than 2xAD/MTNR- mice (*p = 0.02). MEL treated 16 month mice had higher complex IV activity in both NonAD/MTNR+ (*p < 0.001) and NonAD/MTNR- (*p < 0.001) groups. MEL treatment prevented the increased complex IV activity in 2xAD/MTNR+ mice (*p < 0.001). MEL treatment of NonAD/ MTNR- mice did not restore complex IV activity to the level present in NonAD/ MTNR+ mice (*p < 0.05). Evaluated with 2-way ANOVA; error bars represent SEM

Decreased cytochrome c oxidase (complex IV) activity is a hallmark of brain aging. Therefore, we performed complex IV activity assays on striatal extracts on MTNR+ and MTNR- mice at 13 (Fig. 9a) and 16 months (Fig. 9b) of age. There was a striking 42 % decline in COX activity during this time period in extracts from MTNR+ mice (p < 0.001) and a 56 % decline in extracts from MTNR- mice (p < 0.001). When MTNR- and MTNR+ mice were compared, there was a trend for decreased complex IV activity at 13 months of age (p = 0.14) and a significant 33 % reduction in complex IV activity in MTNR- mice by 16 months of age (p = 0.005). For a reference point in young adult mice, we also measured complex IV activity at 6 months of age (data not shown). While there was no drop in complex IV activity between 6 and 13 months of age in MTNR+ mice, there was a 34 % drop in complex IV activity during this time in MTNR- mice (p <0.001). Therefore from 6 to 16 months of age, we found that MTNR- mice showed 33 % more loss of complex IV activity than MTNR+ mice (67 % loss in MTNR- mice compared to 50 % loss in MTNR+ mice). So the presence of MEL receptors slightly delays the loss of complex IV activity with normal aging.

#### MEL-mediated prevention of the aging-induced loss of complex IV activity is mostly independent of MEL receptors

As has been shown by others with aging [45] or hypoxia [46] in rats, we found that MEL treatment completely blocked the decline in complex IV activity from 13 to 16 months of age in both MTNR+ (p < 0.001) and MTNR- (p < 0.001) mice (Fig. 9). Therefore, MEL treatment prevents the aging-related decline in complex IV activity through predominately receptor-independent mechanisms, with this being especially evident between 13 and 16 months of age.

#### AD-induced increase in complex IV activity in 16-month mice

Several studies have shown that AβPP^swe^ transgenic mice are characterized by complex IV deficiency [47, 48], as amyloid-beta has been shown to reduce complex IV activity in isolated mitochondria [49]. Unexpectedly, in our experiments (Fig. 9) there was no decrease in complex IV activity in 13-month 2×AD mice when compared to NonAD and even an increase in complex IV activity from 13 to 16 months of age (p = 0.01). The reason for this unexpected finding is likely the hybrid vigor of the outbred strain or the examination of striatum instead of hippocampus or cerebral cortex, the brain regions most severely effected in AD. In either case, we effectively used this increased complex IV activity in the 16 month AD mice as a marker of an AD-induced mitochondrial stress response when examining the effects of MEL receptor knockout and MEL treatment. Strikingly, the unaltered complex IV activity in the 2×AD mice at 13 months of age parallels the lack of behavioral and cognitive changes observed in these mice at 12 months of age. Likewise, the large changes in complex IV activity at 16 months of age correlates well with the cognitive performance decline by 15 months of age in the 2xAD animals.

At 13 months of age in MTNR- mice, as with MTNR+ mice, the presence of Alzheimer’s proteins did not alter complex IV activity (2×AD/MTNR- vs. NonAD/MTNR-). Also similar to the results observed with MTNR+ mice, a large (45 %) increase in complex IV activity from 13 to 16 months occurred when comparing NonAD/MTNR- vs. 2×AD/MTNR- mice (p < 0.001). 2×AD/MTNR- mice had roughly 26 % decreased complex IV activity at both 13 months (p = 0.03) and 16 months (p = 0.02) of age when compared with the aged matched 2×AD/MTNR+ controls. However, the direction and magnitude of the change in the complex IV activity due to Alzheimer’s protein expression were similar in the 2×AD/MTNR- and 2xAD/MTNR+ mice.

#### Melatonin treatment reduces the high complex IV activity in 2xAD/MTNR+ mice

Surprisingly, MEL treatment greatly decreased complex IV activity in the 2×AD/MTNR+ mice, 30 % at 13 months (p = 0.03) and 52 % at 16 months (p < 0.001). This is the only group where MEL treatment decreased complex IV activity (Fig. 9). This MEL-mediated decrease in complex IV activity in the 2×AD mice is consistent with a mechanism in which there is a reactive oxygen species (ROS)-mediated increase in complex IV activity in 2×AD mice that is prevented by melatonin treatment. In the 2×AD/MTNR- mice, the complex IV activity was lower than in 2xAD/MTNR+ mice and melatonin treatment did not have a statistically significant effect on the activity.

### Antioxidant transcript analysis by qPCR

**Fig. 10:**
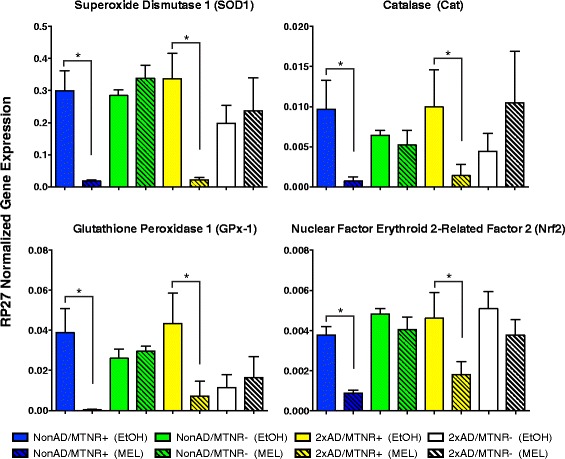
Melatonin treatment effect on anti-oxidant transcript expression in frontal cortex of 13-month old mice. Melatonin significantly (*p* < 0.05) lowered transcript levels for four markers of the antioxidant response system in a receptor-mediated fashion. This effect was seen in both NonAD and 2xAD mice that possessed melatonin receptors (MTNR+), but not in mice that lacked these (MTNR-). Evaluated with 2-way ANOVA; *n* = 4–6; error bars represent SEM

For tissues collected at 13 months, expression levels of superoxide dismutase 1 (SOD1), glutathione peroxidase 1 (GPx-1), and catalase (CAT) were determined in the frontal cortex by quantitative PCR. Additionally, we measured the transcript levels of nuclear factor erythroid 2-related factor 2 (Nrf2), a transcription activator that binds to antioxidant response elements in many genes involved in the cellular response to oxidative stress. Figure 10 shows that in both NonAD and 2xAD mice, the very marked effects of MEL versus vehicle (0.1 % ethanol) on antioxidant signaling in MTNR+ mice are notably absent in MTNR- mice.

## Discussion

Transgenic mice models for studying the cellular and molecular basis of AD have been used for many years, but rarely for investigations into the therapeutic potential of MEL to slow the progression of this disease [24, 50, 51]. Our 2009 publication [23], detailed the neuroprotective effects of long-term oral MEL administration (~0.5 mg/day) on cognitive performance, brain Aβ levels/deposition, and antioxidant enzyme expression in the AβPP^swe^/PSEN1dE9 (2xAD) mouse model. Subsequent recent studies continue to demonstrate that MEL has significant and reproducible prophylactic properties in several mouse models of AD [33, 52, 53].

The primary goal of the present study was to determine the role of the cognate G-protein coupled melatonin receptors in mediating the neuroprotective action of MEL in the 2xAD mouse model. As reported by the supplier (Jackson Laboratories) the 2xAD animals begin developing cognitive deficits after 12 months [54]. We saw no significant cognitive deficits at 12 months of age when using three common behavioral tests (data not shown). However, at 15 months the picture was markedly different. Using the novel object recognition test (NORT), which assesses short-term memory in a non-spatial task, the cognitive protection afforded the AD mouse brain by MEL was equivalent in both the mice with MTRs as well as in the KO mice lacking MTNRs (Fig. 3). Whereas both vehicle-treated AD groups performed poorly (i.e. they were unable to recognize novelty), the MEL-treated 2xAD mice were able to perform at the level of the non-transgenic (NonAD) vehicle-treated control mice irrespective of whether they were with or without the melatonin receptors. These results are consistent with the view that MEL preserves non-spatial cognitive performance in 2×AD mice even in the absence of G-protein coupled membrane MTNRs.

Additional cognitive testing was conducted with the circular platform (Barnes) test for spatial reference learning and memory (Fig. 4). In this test, 15 month-old 2xAD mice without melatonin receptors that had received MEL had lower latencies to the escape hole on day 1, while the 2xAD mice with receptors learned to be even faster over the course of the 8-day testing paradigm when compared to vehicle-treated controls (Fig. 4a). The day 1 differences likely reflect enhanced long-term memory in MEL-treated mice, as the same animals were tested earlier at 12 months of age. Fig. 4b depicts comparative performance on the Barnes test between 12 months and 15 months of age. Significant improvements with age were seen in the 2×AD mice that had received MEL, with more pronounced improvements in the MTNR+ mice.

Mice were also tested with the Morris Water Maze, which includes elements of spatial learning as well as assessment of working memory. As would be expected, the NonAD/MTNR+ mice performed well in both domains (Fig. 5), while the vehicle-treated 2×AD groups performed poorly on both. Notably, cognitive performance of the 2xAD/MTNR+ mice that received MEL was similar to the NonAD/MTNR+ control mice for both testing components. 2×AD/MTNR- mice that received MEL did not show neuroprotection, as their performance levels were comparable to the vehicle-treated 2×AD/MTNR- mice. Thus, the results of these cognitive tests confirm that MEL is capable of preserving memory in 2×AD mice in both non-spatial and spatial domains, as shown previously by several research groups [23, 25, 40]. Additionally, our new data reveal that MEL effects are dependent on the MTNRs in the case of hippocampal-dependent spatial learning tasks, such as the Barnes Maze and the Morris Water Maze, but independent of MTNRs for non-spatial learning.

Acute MEL administration has been reported to have anxiolytic effects in both animal models [55, 56] and in human trials [57, 58]. In addition, Ochoa-Sanchez and colleagues [59] reported that the novel MT2- selective agonist, UCM765, showed anxiolytic properties in the Open Field test and Elevated Plus Maze. Recently, Di Paolo et al. [60] reported that MEL had anxiolytic effects in both NonAD and Tg2576 (AβPP^swe^) mice. In the present study, we could confirm that the anxiolytic effects of MEL depended on the presence of the MTNRs (Fig. 2). MEL trended to influence anxiety in the 2xAD mice, but only in the presence of melatonin receptors.

For the sake of completeness we also assessed sensorimotor function at 12 and 15 months of age in 2xAD and control mice receiving MEL. No evidence of sensorimotor deficits was seen on the Rota-rod or Platform Recognition tests at any of the ages tested (Fig. 1) and there were no statistical differences between any genotypes or treatment groups. The slight improvements in both tests at 15 months vs. 12 months are likely to be due to the effects of experience. Furthermore, despite their C3H background, these mice clearly had no visual deficits, as was confirmed by the behavioral performance on various tests requiring spatial learning (see Figs. 4 and 5). Evaluation of open field activity at 12 months and at 15 months of age revealed no significant differences in any groups (Fig. 1). The slightly increased activity levels seen in the 2xAD/MTNR- mice at 12 months is likely a consequence of genetic deletion of the MTNRs, which we have recently demonstrated to be associated with mild hyperactivity [42].

As a neuropathological correlate of AD progression in these animals we assessed amyloid plaque load by immunohistochemistry in the hippocampi and frontal cortex of 15 month-old mice. A significant reduction of plaque area was seen in MEL-treated mice as compared to vehicle (Fig. 7), with MTNR+ mice that received MEL having 33.5 % less plaques than controls (P < 0.001), while MTNR- mice that received MEL having 18 % less plaques than controls (P = 0.0034), thus comprising the majority of the protective effect of the MEL treatment on plaque deposition. Identical plaque loads were seen in control 2xAD mice, indicating that the genetic deletion of the melatonin receptors does not predispose the animals to more severe neuropathology as they age. Indeed, in our recent comprehensive characterization of the NonAD mice [42] we demonstrated that the MTNR- genotype slightly, but significantly, did just the opposite, i.e. it enhanced motor performance, cognitive function and long-term potentiation as measured in a hippocampal slice preparation. The plaque results from the current study (Fig. 7) confirm that the neuroprotective effects of MEL are largely independent of melatonin receptors, although maximal protection was achieved when melatonin receptors are expressed. In other words, both receptor signaling pathways as well as direct intracellular effects of MEL appear to contribute to its plaque-inhibiting mechanism of action with direct, receptor-independent actions predominating.

Although not the central focus of the current investigation, it is perhaps worthy to note that the 2×AD mouse model has been described by the commercial supplier as having a significant degree of mortality due to seizures in early life [61]. While we saw no evidence of seizures for the mice expressing the melatonin receptors, we did notice that the 2×AD mice without melatonin receptors succumbed in significant numbers in the first 6–9 months of life (Fig. 6). Brains from these animals showed no signs of plaque formation at 4 months and furthermore, at the completion of the study cognition in these animals with melatonin treatment was similarly protected as in the 2xAD/MTNR+ mice (Figs. 4 and 5). Administration of MEL to this group only marginally slowed the loss of the 2xAD/MTNR- mice. It is not entirely clear why the genetic deletion of the melatonin receptors is associated with poor early survival in the 2×AD mice, although an alteration in the balance between inhibitory and excitatory circuits in mice lacking the melatonin receptors [42] might be a possibility worth further testing.

The processing of the amyloid precursor protein in the AD brain generates the pathogenic peptides, Aβ_1–40_ and Aβ_1–42_, with the latter thought to be the major neurotoxic species [62, 63]. Although the blood amyloid levels in this mouse model of AD reflect both central and peripheral APP processing and clearance, we felt that as an indirect measure of MEL’s potential to alter these activities, it would be informative to assess plasma levels of these peptides in our 2xAD mice during the course of plaque development. Thus, using commercial ELISAs we determined plasma levels of Aβ_1–40_ and Aβ_1–42_ at ages 9 (cognitively presymptomatic) and 14 months (Fig. 8). At 9 months of age – when plaques are beginning to accumulate – treatment of 2xAD mice with MEL led to lower plasma levels of Aβ_1–40_ and Aβ_1–42_ in the MTNR+ mice and a similar trend in the MTNR- mice. By 14 months of age (by which time cognitive symptoms are fully expressed), MEL-treated 2xAD mice of the MTNR+ genotype had a significant reduction in plasma Aβ_1–42_ as well as the Aβ_1–42_/Aβ_1–40_ ratio (data not shown), concomitant with significantly lower amyloid load in the brain (Fig. 7). In view of evidence that Aβ_1–40_ and Aβ_1–42_ may be cleared from the brain by different mechanisms [64, 65], our overall results are consistent with a general reduction of amyloidogenic APP processing (rather than Aβ clearance) in response to MEL administration. In agreement with this hypothesis [25] measured hippocampal levels of Aβ_1–40_ and Aβ_1–42_ in the Tg2576 AD mouse and reported significant reductions following treatment with MEL. Additionally, this group demonstrated that MEL treatment decreases Aβ processing via reductions of hippocampal presenilin and β-secretase levels. More recently, Shukla et al. 2015 reported that MEL upregulates alpha-secretase protein levels and subsequent catalytic cleavage of beta-APP to nonamyloidogenic products in cultured neuronal and non-neuronal cells [66]. Thus, our current results showing melatonin receptor-independent MEL-induced decreases in plasma Aβ_1–42_ levels (Fig. 8), concomitant with protection of non-spatial cognitive performance (Fig. 3) and reduced amyloid plaque load following MEL (Fig. 7) are consistent with the hypothesis that MEL’s neuroprotective activities are at least in part due to its long-term effects on Aβ generation.

Brain mitochondrial function, which is very sensitive to oxidative stress and is impaired in AD [67–69], is protected by MEL through numerous mechanisms. For example, we reported recently that chronic MEL treatment protected in a dose-dependent manner against Aβ-mediated mitochondrial dysfunction at multiple levels in 2xAD mice and that this protection could be blocked by use of specific melatonin receptor antagonists [33]. As a further exploration of this phenomenon, we assessed the brain activity of the mitochondrial enzyme, cytochrome c oxidase (complex IV), in 2xAD mice possessing or lacking MTNRs. Striatal samples were assessed, as all of the hippocampal and cortical tissues from these animals were used for other purposes. However, in our previous study [47] we found that mitochondrial respiratory activity in all three of these brain regions of 2xAD mice were similar.

Unexpectedly, we found increased complex IV activity caused by AβPP^swe^/PS1 expression from 13 to 16 months of age, not decreased expression as we and others have observed when studying AβPP^swe^ Alzheimer’s mice of different genetic backgrounds [33, 70]. We have confidence in our complex IV activity measurements in this report as it is well established that there is an aging-related decline in complex IV activity in rodent brain [71]. This increased complex IV activity in 16-month 2xAD mouse brain may be mediated by mitochondrial proliferation or increased expression of oxidative phosphorylation complex subunits in response to oxidative stress [72, 73]. If the oxidative stress is mild or of short duration, mitochondrial proliferation or increased expression of oxidative phosphorylation complex subunits may increase energy reserves to maintain cellular homeostasis, but when the oxidative stress is high or chronic such as in AD, mitochondria become damaged and these compensatory mechanisms become ineffective in restoring energy levels leading to cell and tissue dysfunction. The hypothesis that the increased complex IV activity in the 2xAD mice is caused by oxidative stress is supported by the observation that MEL treatment of the 2xAD/MTNR+ mice completely prevented the increased complex IV activity. MEL likely accomplishes this by both directly scavenging ROS and by decreasing the production of ROS from the electron transport chain [74]. Melatonin can reduce electron leakage from complexes I and III of the ETC. This can importantly lead to reduced formation of nitric oxide [75], peroxynitrite, and peroxynitrite-derived free radicals such as hydroxyl radicals and nitrogen dioxide, which prevent increased NADPH oxidase and iNOS activities, with an overall effect of decreasing neural inflammation [29].

Consistent with our current findings of increased complex IV activity in the 2×AD mice, another group also found increased complex IV activity in AβPP-expressing mice [76]. They found increased COX activity in the ventral striatum of AβPP23 mice partially backcrossed onto a C57BL/6 background. This report showed increased complex IV activity only in specific regions of the brain. In another report, complex IV activity increased in Tg2576 mice at 5 months of age compared to controls [77]. Another group found similar results with these mice at 7 months of age [78]. Consistent with both of these reports, Tg2576 mice show upregulation of mitochondrial electron transport genes [79]. Increased complex IV activity has also been found in neurons from Alzheimer’s patients [80]. This increased electron transport chain complex activity is consistent with the Inverse Warburg Hypothesis of Alzheimer’s disease, which states that aged, energetically stressed neurons attempt to upregulate oxidative phosphorylation to use the lactate produced by adjacent astrocytes as a respiratory substrate to maintain cellular ATP levels [81]. However, several other groups have measured decreased complex IV activity in various brain regions of Tg2576 mice [70, 82–84]. In addition, reduced complex IV activity has also been measured in double and triple transgenic mouse models of AD combining overexpression of presenilin-1 and/or tau with mutant APP overexpression [85, 86].

A recent report has shown that the Aβ peptide directly inhibits complex I activity of the ETC. [87]. The inhibition of complex IV activity was found to be an indirect result from damage of the mitochondrial phospholipid cardiolipin, required for complex IV activity. Cardiolipin was found to be oxidized as a result of the complex I inhibition, and not from a direct inhibition of complex IV by Aβ as has previously been suggested [49]. Cardiolipin peroxidation is most frequently catalyzed by the cardiolipin peroxidase activity of a cytochrome c-cardiolipin complex [88]. Adding back cardiolipin to aged mitochondria has been shown to restore complex IV activity [43]. In addition, Aβ has been shown to decrease transcription of specific complex IV subunits in certain cell types that may also contribute to the decreased complex IV activity [89]. In the 2xAD mice used in this report, altered electron transport chain function in response to increased amyloid-beta levels may have led to decreased activity of the cardiolipin peroxidase activity of cytochrome c, preserving or even increasing cardiolipin levels resulting in the increased complex IV activity measured.

Another possible reason as to why we were able to measure increased complex IV activity in the 2xAD mice is because we used brain extracts where increased mitochondrial proliferation can be detected, in contrast to when measurements are made using isolated mitochondria where increased mitochondrial proliferation is likely missed unless paying close attention to the mass of mitochondria isolated. It is also possible that complex IV activity was upregulated in the 2xAD brain without increases in mitochondrial proliferation. This could be accomplished through expression of alternative complex IV subunits [90] or through post-translational modification of the complex [91]. When damaged cardiolipin limits complex IV activity slowing the rate of electron transport, oxygen has more time to bind the electrons producing superoxide. Under these conditions increasing complex IV activity would be an effective strategy to increase the rate of electron transport to increase ATP levels while also decreasing mitochondrial superoxide production. In summary, our data show that MEL administration protects MTNR+ mice from AD-induced upregulation of complex IV activity. However, MEL administration did not completely restore the increased complex IV activity in the 2xAD/MTNR- mice suggesting both receptor-dependent and independent effects are important for protection. It is of interest to perform similar experiments using additional brain regions and a different genetic background of mice to determine if these restorative effects of MEL are observed when complex IV activity declines as a result of APP_swe_ expression.

Substantial data have accumulated that link oxidative stress to AD pathogenesis [92–94]. It is thus significant to note that numerous studies have reported MEL effects on markers of oxidative stress [95–98]. Acutely, MEL has been reported to elevate in many tissues the expression of antioxidant enzymes as part of a defense mechanism against free radicals [14, 99]. Previously, we have shown significant effects of long-term MEL on SOD1, GPx-1, and CAT mRNA expression in the brain of 10-month-old 2xAD/MTNR+ mice [23]. Also, in the present study, we evaluated antioxidant expression at 13 months of age. As seen in Fig. 10, long-term MEL treatment was associated with lower mRNA expression of Nrf2, SOD1, GPx-1 and CAT in the brain (frontal cortex) of 13 month-old mice in both 2xAD and age-matched NonAD mice. A lower expression of antioxidant mechanisms is the logical outcome for brains that have reduced levels of oxidative stress due to chronic MEL treatment. These results are entirely consistent with our previous study [33] demonstrating MTNR-dependent effects of MEL in the 2xAD brain. Thus, in view of our current data demonstrating that the effects of MEL on some cognitive functions and on amyloid pathogenesis in 2xAD mice are independent of MTNRs (Figs. 3, 7, and 8), the clear implication of our current study is that the potent effect of MEL on antioxidant gene expression in the 2xAD brain is not the key mechanism for its remarkable neuroprotective capabilities.

## Conclusions

Melatonin has been shown to have reproducible and significant protective properties in a variety of neurodegenerative diseases. While often assumed to involve receptor signaling through one or both of the MTNRs, a direct test of this assumption with animals completely lacking their MTNRs has never been reported. In the current study with 2×AD mice we have demonstrated that in some cognitive domains (spatial learning and memory) MEL neuroprotection is indeed receptor-dependent, while in others (non-spatial learning and memory) it is receptor-independent. In addition, hippocampal and frontal cortical amyloid plaque loads and plasma Aβ_1–42_ levels were significantly reduced by MEL in a receptor-independent manner, while MEL reduced cortical antioxidant gene expression in a receptor-dependent manner. These findings demonstrate that long term MEL significantly reduces AD neuropathology and some associated cognitive deficits in a manner that is independent of antioxidant pathways. Future identification of direct molecular targets for MEL action in the brain should open new vistas for the development of future AD therapeutics.

## Materials and methods

### Animals

All mice were housed and handled in accordance with Federal animal welfare guidelines and in compliance with the Public Health Service Policy on Humane Care and Use of Laboratory Animals (2002) and the Guide for the Care and Use of Laboratory Animals (8^th^ Edition). Experiments were reviewed and approved prior to being carried out by the Institutional Animal Use and Care Committee (IACUC) of the Florida State University (Protocols #1016, 1135; Association for Assessment and Accreditation of Laboratory Animal Care International accreditation unit #001031; Office of Laboratory Animal Welfare Assurance #A3854-01).

The AD double mutant transgenic mice with a C57BL/6 x C3H background were obtained from the Jackson Laboratory (stock # 004462), into which two mutant transgenes were inserted at a single locus. One transgene encodes a chimeric human/murine amyloid beta (A4) precursor protein (AβPP^swe^), which was then further modified to encode the Swedish mutations K595N/M596L found in human. The other transgene encodes the "DeltaE9" mutation of human presenilin 1 which is a deletion of exon 9 and corresponds to a form of early-onset Alzheimer's disease. The AβPP^swe^/PS1 transgenic mouse model (2xAD) was maintained in a hemizygous colony.

We also used a progeny of a University of Massachusetts Medical School mouse colony, where the melatonin receptor knockout strains were generated by a genetic mutation introduced by homologous recombination [100, 101]. Briefly, sites within exon 1 of both the melatonin type 1 receptor gene (MT_1_) and the melatonin type 2 receptor gene (MT_2_) were replaced with phosphoglycerate kinase promoter (PGK-neomycin) cassettes. These C57BL/6 animals were then backcrossed for 10 generations to C3H/He mice because others have used the C3H strain extensively in examining circadian behavioral responses to melatonin, as the C3H strain has rhythmic melatonin production [102] unlike most other inbred strains of mice, including the C57BL/6 [103, 104]. The resultant mice were C3B6 (C3H/He + C57BL/6) melatonin receptor homozygous double knockout mice (MT_1_^−/−^/MT_2_^−/−^), referred to here as MTNR- mice, and their non-transgenic wild-type counterparts, referred to here as MTNR+ mice.

The AβPP^swe^/PS1 heterozygous mice were outcrossed to the melatonin receptor double knockout mice or their non-transgenic, wild-type counterparts to obtain mice that that either had or did not have the AβPP^swe^/PS1 mutations and had or did not have their melatonin receptors. Henceforth, we use the following nomenclature: 2xAD mice without their melatonin receptors are referred to as 2xAD/MTNR- and the 2xAD mice possessing their two melatonin surface receptors are termed 2xAD/MTNR+. The mice without the AβPP^swe^/PS1 mutations are termed “NonAD” and therefore, have the following designations: NonAD/MTNR- for the mice without the AD mutations and without their melatonin receptors, but NonAD/MTNR+ for mice without the AD mutations, but possessing their melatonin receptors. This last group is the complete wild-type, non-transgenic control group. We then administered either melatonin (100 μg/ml; ~0.5 mg/day; Sigma M5250) or vehicle (0.1 % ethyl alcohol; EtOH; Electron Microscopy Sciences 15058) in the drinking water to mice beginning at 4 months of age. On the basis of a human equivalency conversion [105] our 0.5 mg daily dose per mouse corresponds to a daily human dose of 150 mg (for a 75 kg individual). Melatonin stocks were made fresh weekly in EtOH at 100 mg/ml, then diluted 1:1000 in distilled water. The water bottles were darkened to prevent the melatonin from enhanced degradation due to light exposure. Multiple cohorts of the four genotypes were treated and behaviorally tested at 12 or 15 months of age and then sacrificed for tissue collection.

The animals were housed individually in a polycarbonate cage (Ancare; 19 cm x 29 cm x 13 cm) with hardwood laboratory bedding chips (Nepco Beta Chip®), nesting material (Ancare Nestlet), and a polycarbonate mouse igloo (Bio-Serv) for enrichment. They were maintained under a 12 h light–dark cycle (7 am to 7 pm) at 21.1 °C and given *ad libitum* access to LabDiet® 5001 Rodent Chow and water.

### Genotyping by PCR

At approximately 21 days of age, an IACUC and veterinarian recommended inhalation anesthetic, isoflurane, (Butler Schein; 029405) was used to sedate the animal. Once anesthetized, 2 mm terminal segment of tail was removed with a sterile scissors. Hemostasis was achieved using a silver nitrate applicator stick (Butler Schein; 005383) and potential pain and discomfort were alleviated by applying bupivacaine hydrochloride in sterile isotonic solution (Sigma B5274; 2.5 μg/ml) locally to the excision site for long-acting pain management. Post-surgical excision site monitoring occurred for 10 days. The tail biopsy was placed in 250 μL of DirectPCR Tail lysis buffer (Viagen 101-T) with 10 μL Proteinase K solution (Viagen 501-K) and lysed for 6 h at 55 °C. The sample was then incubated at 85 °C for 45 min and briefly centrifuged. The supernatant containing the genetic material was collected and stored at −20 °C for subsequent PCR analysis. The resulting PCR products were separated by gel electrophoresis on to a 1.5 % agarose gel (EMD Millipore OmniPur® 2120-OP) for 25 min at 110 volts and subsequently imaged on a Bio-Rad Gel Doc™ XR. Confirmation of genotypes for the MT1 ^−/−^ / MT2 ^−/−^ double knockout (MTNR-) mice, the AβPP^swe^/PSEN1dE9 (2xAD) mice and their non-transgenic (NonAD/MTNR+) wild-type counterparts was performed. Subsequently, RNA-Sequencing was performed on mouse frontal cortex and hippocampal samples per previously published protocols [106]. Sequencing was performed on an Illumina HiSeq 2500 and confirmed that no melatonin receptor expression was present in the MTNR- mice, while both MT1 and MT2 expression were clearly evident in MTNR+ samples. The RNA-Sequencing data were confirmed by qPCR for both MT1 and MT2 [42]. Primers used for genotyping are listed in Table 1.

**Table 1:** Gene-specific primers used for qPCR experiments and genotyping

Gene	Gene-specific Primers 5’ – 3’	GenBank# or Primer ID	Amplicon Size	Anneal Temp
SOD1	S: ATG GCG ATG AAA GCG GTG TG	NM_011434	460	59 °C
	AS: GCG CAA TCC CAA TCA CTC CA			
GPx-1	S: ATG TGT GCT GCT CGG CTC TC	NM_008160	590	60 °C
	AS: TGC TGG GAC AGC AGG GTT TC			
CAT	S: AGG TTT GGC CTC ACA AGG AC	NM_009804	239	58 °C
	AS: GCG GTA GGG ACA GTT CAC AG			
Nrf2	S: AAC GAC AGA AAC CTC CAT CTA C	NM_010902	94	57 °C
	AS: AGT AAG GCT TTC CAT CCT CAT C			
Rp27	S: CCA GGA TAA GGA AGG AAT TCC TCC TG	NM_024277	297	59 °C
	AS: CCA GCA CCA CAT TCA TCA GAA GG			
MT1R	GAG TCC AAG TTG CTG GGC AGT GGA	mMT1R-REV-WT		94 °C
	GAA GTT TTC TCA GTG TCC CGC AAT GG	mMT1R-FW-WT	480	
	CCA GCT CAT TCC TCC ACT CAT GAT CTA	mMT1R-NEOFW-KO	240	
MT2R	CCA GGC CCC CTG TGA CTG CCC GGG	mMT2R-FW-WT		68 °C
	CCT GCC ACT GAG GAC AGA ACA GGG	mMT2R-REV-WT	272	
	TGC CCC AAA GGC CTA CCC GCT TCC	mMT2R-NEO-REV	550	
AβPP	AGG ACT GAC CAC TCG ACC AG	oIMR3610	377	52 °C
	CGG GGG TCT AGT TCT GCA T	oIMR3611		
Psen1	AAT AGA GAA CGG CAG GAG CA	oIMR1644	608	54 °C
	GCC ATG AGG GCA CTA ATC AT	oIMR1645		
Pos Ctrl	CTA GGC CAC AGA ATT GAA AGA TCT	oIMR7338	324	54 °C
	GTA GGT GGA AAT TCT AGC ATC ATC C	oIMR7339		

### Behavioral testing

Mice were tested in a behavioral battery, which included open field activity, elevated plus maze, rota-rod, platform recognition, Barnes maze, Morris water maze, Probe test, and novel object recognition test. For all behavioral testing, testers were blinded to the animal identification number and their respective genotypes and were only allowed to know the artificial 5-digit alphanumeric scheme created to identify the animals. Behavioral tests were performed during the 12-h dark phase, corresponding to the active phase for mice, under white light conditions from 7 pm to midnight. Video recording and/or computer monitoring was utilized to provide accurate analysis of results at a later date. Behavioral testing was conducted with mice at either 12 months of age (2 cohorts; *n* = 12–14) or at 15 months of age (1 cohort; *n* = 6–7). Any mice tested at 15 months of age were also tested at 12 months of age. Animals of all genotypes (2xAD/MTNR+, 2xAD/MTNR-, NonAD/MTNR+, NonAD/MTNR-) and treatments (melatonin, vehicle treated) animals were tested at the same times in the same testing apparatus. In general, NonAD mice did not show vehicle (EtOH) versus MEL treatment differences and are therefore not shown. Any cognitive differences between NonAD/MTNR+ and NonAD/MTNR- animals have already been described in our previous publication [42].

#### Sensorimotor and locomotor behavior

An accelerating *Rota*-*rod* treadmill (Med Associates, Inc., St. Albans, VT, USA) was used to assess motor and coordination deficits. In this test, the mouse was placed on a rotating horizontal rod, which was 3.2 cm in diameter. In each trial, the animal was placed on the rod and allowed to acclimate while the rod rotated at a constant 4 rotations per minute (rpm). Subsequently, the speed was accelerated from 4 to 40 rpm. Latency in seconds was recorded when either the mouse dropped to the platform (16.5 cm) or until 5 min elapsed.

*Platform Recognition* is a swimming-based sensorimotor task often used to measure the animal’s ability to visually identify/recognize a variably placed elevated platform, and its ability to approach and ascend on to the surface of the platform. Specifically, this test was utilized as a means of determining if any animal had visual deficits that could impair their ability to perform in later cognitive tests, as mice on a combined C3H background can have retinal degeneration. The test was conducted in a pool that measured 88 cm in diameter and 21 cm deep and was separated into four equal quadrants by lines suspended above the pool. The temperature of the water was held at 26 °C. A 9 cm circular platform with a prominent black ensign attached was raised 0.8 cm above the surface of the water. The platform had a textured surface to aid the mouse in climbing onto the surface and to encourage the mouse to stay on the platform. A 61 cm high circular barrier was placed around the pool to lessen the escape of the mice. The mice were given four, 60-s maximum trials per day for four days. On each day, the first two trials were consecutive and were separated by a 30-min gap before the next two consecutive trials were performed. The mice began each trial facing the wall of the pool in the same quadrant for each trial for that particular day. The platform was placed in a different quadrant at the start of each day and the mice were started from a different quadrant than the previous day. For each trial, the escape latency was recorded in seconds, concluding when the mouse obtained the platform. A 30-s stay on the platform between consecutive trials was encouraged. A full 60 s was recorded for any animal that did not obtain the platform and the mouse was gently guided towards the platform and encouraged to remain on the platform for 30 s. During the 30-min gap between consecutive trials and after daily testing, the mice were washed and placed under warming lamps to dry. For statistical analysis, escape latencies were averaged for the first two trials and the second two trials per day.

An *Open Field activity* test was used to measure exploratory behavior and general activity. The mice were individually placed into an open field box (45 cm long × 43.9 cm wide × 30 cm high). The area was divided into a 4x4 grid (16 squares) with each square measuring 11.8 cm × 11.2 cm. Mice were given one 5-min trial in which they were free to move around the box. Activity was scored as the number of line crossings by the mouse during the trial. A line crossing was defined as all four limbs entering into a new square.

#### Anxiety behavior

To assess anxiety, animals were evaluated using a near-infrared (NIR) backlit *Elevated Plus Maze* (EPM; Med Associates, Inc., St. Albans, VT, USA) consisting of an elevated plus-shaped maze with two opposite open arms (50 cm × 10 cm) and two opposite closed arms (50 cm × 10 cm with 40 cm walls). The tests were conducted with NIR backlight for animal tracking. The task was initiated by placing the test subject into the center of the maze facing an open arm. Activity was monitored via a mounted overhead camera and video tracking system for a 5 min period. Entries into each arm, and the time spent in open arms, closed arms, and center of maze were evaluated.

#### Cognitive behavior

The *Barnes Maze* (Med Associates, Inc., St. Albans, VT, USA) is a type of delayed match-to-place experiment used to assess spatial/reference learning and memory. Visual objects arranged around the maze served as spatial cues. The maze surface was 121.8 cm in diameter and 140.2 cm high with 40 equally spaced holes on the periphery of the surface. A dark box filled with bedding was positioned under one of the holes on the platform to allow the mouse to escape from aversive stimuli. Each mouse was assigned a specific unique escape hole for the duration of the experiment. For each trial, the mouse was placed in the center of the platform facing away from its target escape hole. The aversive stimuli included one high-intensity fan blowing at the level of the platform and two 120-watt flood lamps hung from the ceiling near to and aimed at the platform. Each mouse was given one 600-s trial per day for 8 days and the escape latency in seconds was recorded for each trial. A full 600 s was recorded for any animal that did not find its target escape hole during a trial.

For the *Morris Water Maze*, the mice must adopt a spatial learning strategy rather than a recognition strategy used in the platform recognition test. All of the equipment was set up the same as in the platform recognition with a few exceptions. A circular platform without an ensign was placed 1.5 cm below the surface of the water, which was at 7.8 cm high. The test was run for 6 consecutive days. The platform remained in the same quadrant for each day of the testing. During the training phase (first 5 days) mice were given 4 successive 60 s trials per day. The mice were started from a different quadrant in each of the trials. The same quadrant start pattern was used across the 5 days of testing. Latency to find the platform (a maximum of 60 s) was recorded for each trial and the four daily trials were averaged for statistical analysis. If a mouse did not find the platform in the 60 s allowed they were gently guided towards the platform. Once a mouse was on the platform they were allowed to sit for 30 s. If a mouse found the platform by itself and chose to jump off a new trial began. Animals that did not find the platform were given a latency score of 60 s. The *Probe Test* was the second phase of the experiment and was conducted on day 6. During this phase, the platform was removed and the mice were started from the quadrant opposite of the platform quadrant. The percentage of time spent swimming in each quadrant, including the goal quadrant (which previously held the platform) was recorded.

The tendency of mice to interact more with a novel object than with a familiar object has been used as a method to study learning and memory [107]. The *Novel*-*Object Recognition task* (*NORT*) was performed in a small, opaque open field box (36 cm long by 32 cm wide by 29 cm high). On the day before the experimental sessions, animals were habituated to the experimental room and NORT chamber for 10 min. Twenty-four hours later, mice were given 10 min to interact in the testing chamber with two-identical objects. The animal was removed from the apparatus and given a 1-h training-to-testing interval. One of the now familiar objects was replaced with a novel object, which was previously tested for comparable object manipulability and complexity interactions. The mouse was again placed into the testing chamber and allowed to interact freely with the familiar and novel objects for 5 min. The familiar and novel objects were placed on opposite sides of the testing chamber for each trial and the entire testing apparatus was thoroughly cleaned with 70 % ethanol between each subject. The amount of time spent at both familiar and novel objects were determined via video analysis using a “within object area” scoring method. An animal was scored as interacting with the object when its nose was in contact with the object or directed at the object within ≤ 2 cm. Time spent standing, sitting, or leaning on the secured object was not scored as object interaction.

### Tissue collection for immunohistochemistry

All mice were euthanized by sterile intraperitoneal overdose injection of ketamine (Butler Schein; 100 mg/kg) and xylazine (Vedco; 10 mg/kg) and transcardially perfused first by 0.9 % sterile saline, followed by 4 % paraformaldehyde (PFA). Whole brains were removed and placed into 15 ml of the same PFA fixative used for perfusion for 24 h at 4 °C. Brains were then placed into autoclaved 30 % sucrose solution at 4 °C until the brains sank. They were then sectioned into 40 μm thick coronal slices and placed into a cryoprotectant solution [108] at −20 °C until they were examined by immunohistochemistry.

### Immunohistochemistry

Brain sections were removed from the cryoprotectant solution and washed at room temperature (RT) 3 times for 10 min in 0.1 M phosphate-buffed saline (PBS) with 0.1 % Triton X-100 (PBSX), followed by a 15-min incubation in 1 % H_2_O_2_ (prepared right before use) and then washed an additional 3 times in PBSX. Slices were then blocked at RT for 60 min in 1 % normal goat serum (NGS), and incubated in PBS containing 0.4 % Triton X-100, NGS, and rabbit anti-human amyloid-beta (Aβ; Cell Signaling #2454) primary antibody for 18 h at 4 °C. Initial antibody titration occurred at the following dilutions: 1:1 K, 1:3 K, 1:6 K, 1:10 K, and 1:30 K after which 1:6 K was chosen for the best signal to noise ratio. Tissues were washed in PBSX 3 times for 10 min and treated for 2 h at RT in PBS with 0.4 % Triton X-100 and NGS with a biotinylated goat anti-rabbit secondary antibody (Vector Laboratories, Burlingame, CA, USA) and again, rinsed 3 times in PBSX for 10 min each. Next, they were transferred to an avidin/biotin solution made 30 min prior with a Vectastain Elite ABC Kit Standard (Vector Labs) in PBS containing 0.4 % Triton X-100 for 60 min at RT. Slices were washed twice more in 10-min PBSX washes, followed by a 5-min rinse in sodium acetate buffer (pH 7.5-7.6). Sections were processed for exactly 20 min at RT in a nickel-diaminobenzidine (Ni-DAB) solution, prepared from previously made aliquots of NiSO_4_ and DAB solution, sodium acetate, and H_2_O_2_, followed by another 5-min sodium acetate buffer rinse. Before the slices were mounted on slides, they had 2 final 10-min PBS rinses and allowed to dry. On the following day, the dehydration process included rinses for 1 min each in the following: distilled H_2_O; 50 % ethanol; 75 % ethanol; 95 % ethanol; 2 100 % ethanol rinses. The slides were left to air dry for 1 min before two 5-min washes of Histo-clear (National Diagnostics). Slides were cover-slipped with DPX (distyrene, a plasticizer, and xylene; Electron Microscopy Services, Hatfield, PA, USA) mounting medium and examined under light microscopy with an Olympus MVXIO microscope. Percent area with positive Aβ plaque load was analyzed with Fiji (Fiji is Just ImageJ) software [109].

### Blood plasma collection and ELISA for human Aβ_1–40_ and Aβ_1–42_

Mouse blood plasma was collected for quantitative determination of human Aβ_1–40_ and human Aβ_1–42_ peptides using a solid phase sandwich Enzyme Linked-Immuno-Sorbent Assay (ELISA; Invitrogen Cat# KHB3481, KHB3441). Blood collection occurred between the hours of 10 am and 1 pm. Mice were anesthetized with isoflurane and blood was collected via a puncture to the vascular bed at the back of the jaw where the orbital veins, the submandibular veins, and the other veins draining the facial area coalesce to form the jugular vein. The exuding droplets of blood (~0.3-0.4 ml) were collected into an EDTA tube, after which, a sterile gauze compress was applied to the puncture site for 20 s. This rapid blood collection method is more humane than retro-orbital or cardiac puncture, leaving the animals unaffected with no signs of distress [110]. Whole blood was centrifuged at 2000 g for 3 min to separate the plasma, which was stored at −80 °C until processed by ELISA according to the manufacturer’s protocol.

### Tissue collection for oxygen consumption assay and transcript analysis

All mice were euthanized by sterile intraperitoneal overdose injection of ketamine (Butler Schein; 100 mg/kg) and xylazine (Vedco; 10 mg/kg), followed by transcardial 0.9 % sterile saline perfusion. Brains were quickly removed and placed on an ice-cold plate for dissection. The brain was initially bisected along the sagittal plane and the left hemisphere was dissected for hippocampus, striatum, and frontal cortex. The dissected samples were then flash frozen for subsequent analysis. The right hemisphere was similarly dissected and preserved in QIAGEN RNAlater® RNA stabilization reagent for later mRNA analysis by qPCR.

### Cytochrome C oxidase assay

Cytochrome c oxidase activity was performed using a standard polarographic method similarly as described in [111]. Specifically, the striatum from 13 month old mice was dissected out, frozen, thawed, diced into cubes, and placed into a dounce glass homogenizer along with 2.5 ml of ice cold isotonic buffer (7.5 mM sucrose, 0.1 % BSA, 20 mM HEPES, 1 mM EGTA, 215 mM mannitol, pH 7.2). Homogenization was performed in a dounce tissue homogenizer using four strokes with a tight fitting pestle and then the suspension was spun down at 13000 × *g* for 5 min at 4 °C. The supernatant was removed and the pellet was resuspended in 150 μl of the ice-cold isotonic buffer above with the addition of 1 mM n-dodecyl-β-D-maltoside. Thirty-five μl of the solubilized heavy membrane fraction was transferred into a respiratory chamber (MT200A, Strathkelvin Instruments) containing 315 μl of buffer with 125 mM KCl, 1 μM FCCP, 50 μM cytochrome c, 2 mM ascorbate, 1 μM antimycin A, 5 mM tetramethylphenylenediamine (TMPD), 2.5 mM phosphate, and 20 mM HEPES, pH 7.2. The protein concentration in the respiratory chamber was roughly 2 mg/mL. The rate of oxygen consumption was obtained using a Clark oxygen electrode. After 90 s, 700 μM KCN was added to obtain a non-cytochrome c oxidase rate of oxygen consumption, which was subtracted off. The protein concentration was determined by a BCA protein assay (Pierce) and the respiratory rate was normalized to protein concentration.

### Isolation of mouse brain mRNA and real-time qPCR

Total cellular RNA was extracted from homogenized frontal cortices with QIAShredder™ columns in QIAGEN’s buffer RLT and β-mercaptoethanol in a 100:1 ratio followed by processing with the QIAGEN® RNeasy Kit® and DNase Set treatment kits according to the manufacturer’s protocols. The RNA concentration was measured with a Thermo Scientific Nanodrop photometer. For analysis of transcript levels, one μg of total RNA was reverse-transcribed to cDNA by means of the Bio-rad iScript^TM^ reverse-transcription system. Amplification of the cDNA was performed on a Bio-Rad CFX96 Touch™ Real-Time PCR Detection System using the iQ SYBR® Green Supermix protocol according to the manufacturer specifications. The following thermal cycling parameters were used: initial heat activation of the DNA-polymerase was performed at 95 °C for 5 min. Thereafter, 40 cycles of 95 °C (10 s), 57–60.5 ° C (10 s) and 72 °C (30 s) were run. The primer sequences and annealing temperatures for the gene transcripts that were analyzed are listed in Table 1.

### Statistics

All calculations, comparisons and statistical analysis were performed using GraphPad Prism version 6.0a software for Mac OS X (GraphPad Software, La Jolla, California, USA, www.graphpad.com). The values shown on the graphs represent the means ± S.E.M. from independent experiments. Behavioral tests comparing differences between genotypes and treatments were computed with a 2-way ANOVA followed by Bonferroni’s multiple comparison of means. The Kaplan-Meier survival curve was analyzed with a Wilcoxon-Gehan-Breslow log-rank test (chi square). Tests conducted with multiple trials or time points were analyzed with a 2-way ANOVA for repeated measures and followed by the Bonferroni test for *post hoc* comparison. Statistical values reaching *p* ≤ 0.05 were considered significant.

## Abbreviations

2xAD: Mice with double Alzheimer mutation (AβPP^swe^/PSEN1dE9)
Aβ: Amyloid-beta
AβPP^swe^: Amyloid-beta precursor protein, human Swedish mutation
AD: Alzheimer’s disease
C3B6: Mice with a combination C3H/He and C57BL/6 background
CAT: Catalase
cDNA: Complementary deoxyribonucleic acid
CNS: Central nervous system
DD: Dark-dark or constant dark
DNA: Deoxyribonucleic acid
DPX: Distyrene, a plasticizer, and xylene
EPM: Elevated plus maze
GPx-1: Glutathione peroxidase 1
HEPES: 2-[4-(2-hydroxyethyl)piperazin-1-yl]ethanesulfonic acid
HPC: Hippocampus
IHC: Immunohistochemistry
MEL: Melatonin
mRNA: Messenger ribonucleic acid
MTNR: Melatonin receptor
MTNR-: Mice without melatonin receptors
MTNR+: Mice with melatonin receptors
MTNR1a or MT1: Melatonin receptor type 1
MTNR1b or MT2: Melatonin receptor type 2
MWM: Morris water maze
NGS: Normal Goat Serum
Ni-DAB: Nickel-diaminobenzidine
NonAD: Non-Alzheimer control animals
NORT: Novel object recognition task
Nrf2: Nuclear factor erythroid 2-related factor 2
PBS: Phosphate-buffered saline
PBSX: Phosphate-buffered saline with 0.1 % Triton X-100
PCR: Polymerase chain reaction
PFA: Paraformaldehyde
PGK: Phosphoglycerate kinase promoter
PS1 or Psen1: Presenilin 1
qPCR: Quantitative polymerase chain reaction
SDS-PAGE: Sodium dodecyl sulfate-polyacrylamide gel electrophoresis
SOD1: Superoxide dismutase 1
